# Stable Single-Mode Operation of Distributed Feedback Quantum Cascade Laser by Optimized Reflectivity Facet Coatings

**DOI:** 10.1186/s11671-018-2455-z

**Published:** 2018-02-02

**Authors:** Dong-Bo Wang, Jin-Chuan Zhang, Feng-Min Cheng, Yue Zhao, Ning Zhuo, Shen-Qiang Zhai, Li-Jun Wang, Jun-Qi Liu, Shu-Man Liu, Feng-Qi Liu, Zhan-Guo Wang

**Affiliations:** 1 Key Laboratory of Semiconductor Materials Science, Institute of Semiconductors, Chinese Academy of Sciences; Beijing Key Laboratory of Low Dimensional Semiconductor Materials and Devices, Beijing, 100083 China; 2 College of Materials Science and Opto-Electronic Technology, University of Chinese Academy of Sciences, Beijing, 101408 China

**Keywords:** Quantum cascade laser, Distributed feedback, Facet coating, Stable single mode

## Abstract

In this work, quantum cascade lasers (QCLs) based on strain compensation combined with two-phonon resonance design are presented. Distributed feedback (DFB) laser emitting at ~ 4.76 μm was fabricated through a standard buried first-order grating and buried heterostructure (BH) processing. Stable single-mode emission is achieved under all injection currents and temperature conditions without any mode hop by the optimized antireflection (AR) coating on the front facet. The AR coating consists of a double layer dielectric of Al_2_O_3_ and Ge. For a 2-mm laser cavity, the maximum output power of the AR-coated DFB-QCL was more than 170 mW at 20 °C with a high wall-plug efficiency (WPE) of 4.7% in a continuous-wave (CW) mode.

## Background

Mid-infrared quantum cascade lasers (QCLs) [1] are one of the most promising light sources for many commercial applications. These practical applications such as gas sensing, free-space communication, and high-resolution spectroscopy [2–5] would require QCL with high power, improved single-mode reliability, and low cost. As a result, since the first distributed feedback (DFB)-QCL was demonstrated in 1997 [6], the performance of these devices has been made strong improvements with the demonstration of room temperature continuous-wave (CW) operation with high power across the mid-infrared region [7–10]. However, most DFB-QCLs based on buried grating structure would have the problem of random cleaved facets that determine lasing frequency mode. Due to the same amount of loss in two band-edge modes, stable single-mode operation cannot be guaranteed [11]. Especially under high temperature condition or large injection current, mode hopping always happens which is detrimental for the applications in these single-mode devices.

To get a stable single-mode operation, a quarter-wave phase shift (λ/4 PS) was introduced in grating period so that the laser can work in defect mode; thus, competition between the two band-edge modes can be avoided. But electron beam lithography must be used for the fabrication of λ/4 PS grating, which is time-consuming and expensive [12]. Gain-coupled DFB laser is a good choice to achieve stable single-mode operation for conventional semiconductor laser [13]. However, it is unrealistic for QCL to make the gain-coupled DFB lasers because of the great loss caused by etched active region. Another artful method is to use the cavity loss coupling mechanism for increasing the difference loss between two DFB modes. It is believed that appropriate reflectivity facet coating can achieve stable single-mode operation at even high temperatures and large currents. Although there are some researches devoted to facet coating, they always focus on forming optimal equivalent cavity length *L*_opt_ to preserve wall-plug efficiency (WPE) for lasers rather than the single-mode reliability [14, 15]. Also, the optimized reflectivity coating should be a promising way to solve the competitiveness between two DFB modes and interesting to be investigated systematically.

In this paper, stable single-mode operation of DFB-QCLs at *λ* ~ 4.76 μm is presented after antireflection (AR)/high reflection (HR) coating. The AR coating consists of a double layer dielectric of Al_2_O_3_ (380 nm) and Ge (33 nm). These devices display a very low threshold current density of 0.65 kA/cm^2^ at 20 °C. Single-mode emission with a side-mode suppression ratio (SMSR) above 26 dB is achieved up to a temperature of 90 °C in CW operation without any mode hopping. It is believed that anti-reflectivity coating on the front facet is very valuable for suppressing random phase of cavity facet.

## Methods

### Theory and Simulation

**Fig. 1:**
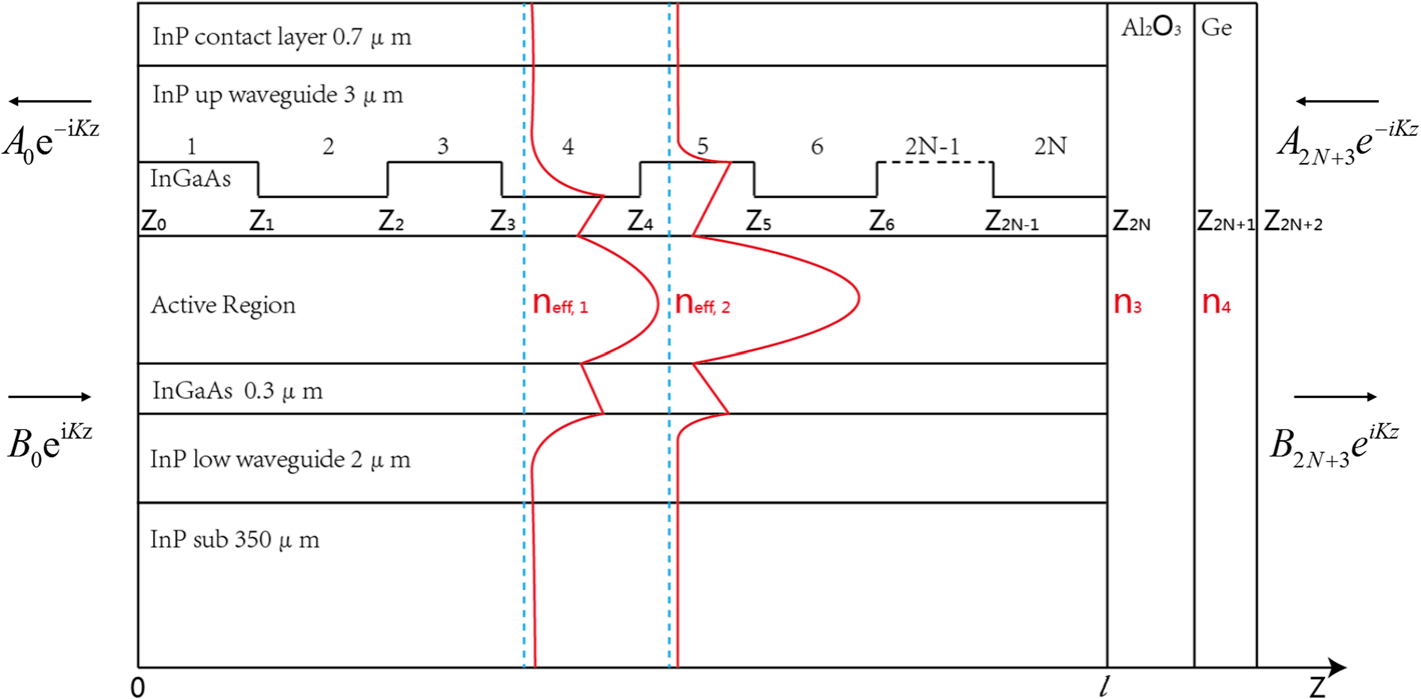
Schematic illustrating the model of finite DFB cavity with antireflection coating on the longitudinal optical mode profile

The core of simulation on antireflection coating effect in finite DFB cavity is the calculation of mode loss for two band-edge modes. Transfer matrix method would be an appropriate way to analyze the whole laser structure [16, 17]. We consider the application of this method to devices which have a longitudinal refractive index profile similar to that shown in Fig. 1. This schematic drawing illustrates the effect of grating with small different effective index perturbation (*n*_eff,1_, *n*_eff,2_) and coated films (*n*_3_, *n*_4_) on the guided mode. The complex refractive indexes of main materials used in calculation are listed as follows: InP (3.088 + i*2e−4), InGaAs (3.4 + i*2.9e−5), active region (3.298 + i*4e−5), high-doped InP (2.81 + i*1.4e−2), SiO_2_ (1.3603 + i*6.3e−4), Au (1.341 + i*32.582), Al_2_O_3_ (1.5348 + i*3.2967e−3), and Ge (4.0165 + i*4e−2). Then, the different effective indexes *n*_eff,1_ = 3.1599 + i*5.17e−5 and *n*_eff,2_ = 3.1662 + i*5.6756e−5 were worked out with COMSOL through partial differential equation (PDE) function. The laser is assumed to be operated in a single transverse mode so that propagation characteristics of light at each point along the laser cavity are described by a single scalar complex quantity, *k*, which is the wave vector of the medium. It is further considered that the laser is linearly polarized and its associated electric field has a sinusoidal time dependence *e*^*i*ω*t*^*.* Following these assumptions given above, a one-dimensional plane electro-magnetic wave factor *E*_z_, which describes the part of special variation of wave function, satisfies the Helmholtz equation

1 $$ \frac{\partial^2{E}_{\mathrm{z}}}{\partial {z}^2}+{K}^2\left(\mathrm{z}\right){E}_{\mathrm{z}}=0 $$

*K*(z) is given by

2 $$ K\left(\mathrm{z}\right)=\frac{\omega }{c}\cdot n\left(\mathrm{z}\right)=k\cdot n\left(\mathrm{z}\right)=\left({k}_{\mathrm{r}}+{ik}_{\mathrm{i}}\right)\cdot n\left(\mathrm{z}\right) $$

where *ω* and *c* are respectively the angular frequency and light velocity and *n*(z) is the complex refractive index at each point along the laser cavity. The wave vector *k* which needs to be solved can be divided into two parts: *k*_r_ and *k*_i_. The real part *k*_r_ determines the wavelength of light in the laser cavity, while the imaginary part *k*_i_ is originated from the mode loss of the finite cavity accounts for attenuation. From Fig. 1, it can be seen that the laser can be considered as a multi-section device with 2*N* + 2 sections where *N* is the grating period. In each of these sections, the electric field *E*_n_(z) is a linear combination of two counter propagating exponentially plane waves where one is decreasing with complex amplitude *A*_n_ and the other is increasing with *B*_n_. The equation is described as follows:

3 $$ {E}_{\mathrm{n}}\left(\mathrm{z}\right)={A}_{\mathrm{n}}\exp \left(-{iK}_{\mathrm{n}}\mathrm{z}\right)+{B}_{\mathrm{n}}\exp \left({iK}_{\mathrm{n}}\mathrm{z}\right) $$

In total, there are 2*N +* 3 interfaces. At each of these interfaces, both the electric field and its derivative with respect to the propagation direction must be equal on both sides of the interface. The equation is obtained as follows:

4 $$ \left[\begin{array}{c}{E}_{2N+3}\left(\mathrm{z}\right)\\ {}{E^{\hbox{'}}}_{2N+3}\left(\mathrm{z}\right)\end{array}\right]=\prod \limits_{n=0}^{2N+2}M\left({d}_n\right)\left[\begin{array}{c}{E}_0\left(\mathrm{z}\right)\\ {}{E^{\hbox{'}}}_0\left(\mathrm{z}\right)\end{array}\right]=\left[\begin{array}{cc}{\mu}_{11}& {\mu}_{12}\\ {}{\mu}_{21}& {\mu}_{22}\end{array}\right]\cdot \left[\begin{array}{c}{E}_0\left(\mathrm{z}\right)\\ {}{E^{\hbox{'}}}_0\left(\mathrm{z}\right)\end{array}\right] $$

The transfer matrix *M*(*d*_*n*_) is given by

5 $$ M\left({d}_n\right)=\left[\begin{array}{cc}\cos \left({kn}_n{d}_n\right)& \frac{1}{kn_n}\sin \left({kn}_n{d}_n\right)\\ {}-{kn}_n\sin \left({kn}_n{d}_n\right)& \cos \left({kn}_n{d}_n\right)\end{array}\right] $$

Considering that the electrically pumped laser is a self-oscillating device, there are no incoming waves from outside the device. This results in the boundary condition *B*_0_ = *A*_2*N* + 3_ = 0, and the equation turns to

6 $$ f= ik{\mu}_{11}+{\mu}_{12}{k}^2-{\mu}_{21}+ ik{\mu}_{22}=0 $$

Each value of the wave vector *k* can be obtained with the aid of Matlab through Eq. (6). The imaginary parts *k*_i_ corresponding to losses of the cavity modes would help to analyze the AR coating effects.

**Fig. 2:**
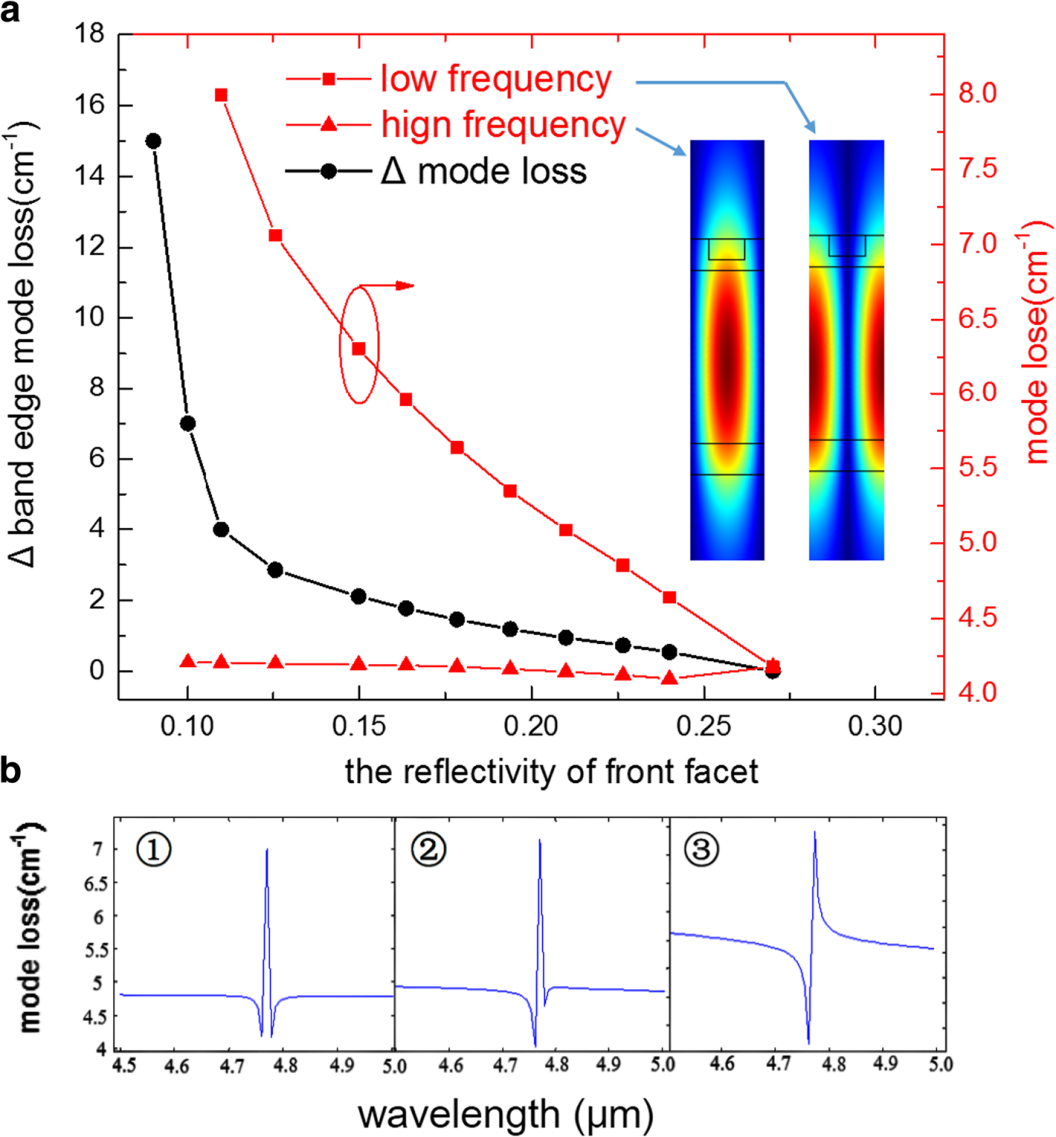
**a** The two red curves are the mode loss of high- and low-frequency mode respectively. The black curve is the differential mode loss between the two band-edge modes labeled as Δ. The inset shows the mode profile calculated for the low- and high-frequency modes, for a single period of the grating. **b** The calculated mode loss spectrum based on transfer matrix simulation with different AR coatings

Figure 2a demonstrates the calculated results based on transfer matrix simulation. As the two red curves have shown, the high-frequency mode loss changes very slowly with the decrease of reflectivity whereas the low-frequency mode increases sharply. The inset shows the mode profile calculated for the low- and high-frequency modes, for a single period of the grating. As plotted, the low-frequency mode has higher electric field magnitude in the grating peaks that is the higher index part of the grating, and also, the high-frequency mode is more concentrated in the lower index part of the grating. For infinite cavity model without facet reflectivity, high-frequency mode always has lower mode loss than low-frequency mode. If the effect of end facet mirrors could be ignored, then the high-frequency mode with the smaller waveguide losses will always lase. However, the presence of end facet mirrors gives reflections that constructively or destructively interfere with the DFB modes in the laser cavity. This interference affects the finite grating-cavity loss of each mode and can determine which mode lases. We note that the effect of the mirrors is largest when the position of both mirrors coincide with a peak in electric field amplitude of one DFB mode, which is also when the mirrors are at a node for the other DFB mode. Here, the mirrors for the uncoated facet coincide with the peak of low-frequency mode, and then, the reflections from the end mirrors maximally constructively interfere with the mode present in the laser cavity. This results in a decreased total mode loss, due to the constructive contribution of the mirror. As the reflectivity decreases and additional phase shift influences by using the double layer AR coating, the loss of low-frequency mode gradually increased with the decrease of reflectivity due to the weakened interference effect and increased mirror loss. Meanwhile, the loss of high-frequency mode changed a little due to the enhanced interference effect. This results in that the Δ mode loss performs as similar as an exponential function especially when the front facet reflectivity is < 0.15. According to the simulation, there exits only one minimum point within whole spectrum when the reflectivity of front facet is < 0.11, which means that mode hop cannot happen in theory because another band-edge mode loss is too high to lase.

Figure 2b shows the three typical mode loss spectrums during simulation where the high- and low-frequency modes are 4.762 and 4.779 μm respectively. The first one is the DFB-QCL without AR coating. We can see the stopband originated from the grating feedback clearly, and the two band-edge modes are almost the same. The second one is specific AR coating with 200 nm Al_2_O_3_ and 5 nm Ge with the reflectivity of 0.22. The differential between two band-edge modes begins to be obvious. The last one shows that with lower reflectivity coating, the Δ mode loss is so big that the low-frequency mode submerged under the loss of stopband. Although the lower reflectivity, the greater Δ mode loss in theory, we should also consider that the extremely low reflectivity causes huge mirror loss in devices which would make the WPE drop sharply. That is a trade-off to choose film thickness based on experiment.

### Device Fabrication

The QCL wafer was grown on an n-doped (Si, 3 × 10^17^ cm^−3^) InP substrate by solid-source molecular beam epitaxy (MBE) based on a two-phonon resonance design. The active core includes 40 stages of strain-compensated In_0.669_Ga_0.331_As/In_0.362_Al_0.638_As quantum wells and barriers, which are similar to Ref. [18]. The layer sequence was as follows: 2-μm lower InP cladding layer (Si, 2.5 × 10^16^ cm^−3^), 0.3-μm-thick matched In_0.47_Ga_0.53_As layer (Si, 4 × 10^16^ cm^−3^), 40 active/injector stages, 0.3-μm-thick In_0.47_Ga_0.53_As layer (Si, 4 × 10^16^ cm^−3^), 3-μm upper InP cladding layer (Si, 2.5 × 10^16^ cm^−3^), and 0.7-μm highly doped InP cladding layer (Si, 5 × 10^18^ cm^−3^). The average doping level of active region was empirically adjusted to 2.4 × 10^16^ cm^−3^. To fabricate the buried grating, the top cladding was removed down to the upper InGaAs layer. The first-order DFB grating with a period of Λ = 0.755 μm (duty cycle *σ* = 20%) was defined on the 300-nm-thick upper InGaAs layer using holographic lithographic technique and subsequently etched to a depth of about 90 nm by wet chemical etching. Then, a 3-μm-thick low-doped (Si, 2.5 × 10^16^ cm^−3^) InP layer followed by a 0.2-μm gradually doped (changing from 1 × 10^17^ cm^−3^ to 3 × 10^17^ cm^−3^) InP layer and a 0.5-μm InP (5 × 10^18^ cm^−3^) contact layer were accomplished in sequence as the upper cladding by metal organic vapor phase epitaxy (MOVPE).

Following implementation of the grating pattern and regrowth, the epi-wafer was etched into 10-μm-wide ridges, and then, the waveguides were reintroduced into the MOVPE system and buried in semi-insulating InP (Fe). A 450-nm-thick SiO_2_ layer was deposited by plasma-enhanced chemical vapor deposition (PECVD) for insulation around the ridge, and electrical contact was provided by a Ti/Au layer. An additional 5-μm-thick gold layer was subsequently electroplated to further improve heat dissipation. The waveguides were cleaved into 2-mm-long bars, and the testing was performed on devices with optimized reflectivity facet coatings. Both rear facet HR coating consisting of Al_2_O_3_/Ti/Au/Ti/Al_2_O_3_ (400/5/100/10/200 nm) and the front facet AR coating consisting of Al_2_O_3_/Ge (380/33 nm) are deposited by e-beam evaporation. The calculated reflectivity of front facet is 3.4% for 4.76-μm wavelength, and the detailed relation between the fluctuation of coating thickness and reflectivity has been discussed in our  previously published paper [19]. The lasers were mounted epilayer side down on SiC heat sinks with indium solder and then wire bonded to an external contact pad. For spectral and electrical characterization, the lasers are mounted on a Peltier element and the temperature was monitored on the heat sink with a thermistor.

## Results and Discussion

**Fig. 3:**
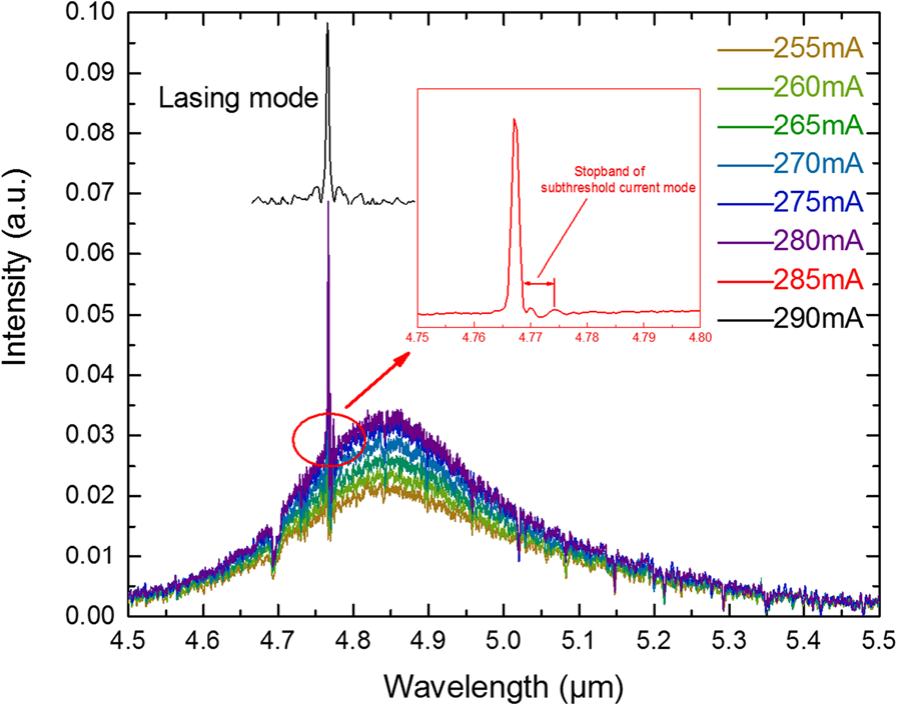
Subthreshold DC spectrum of device measured at 30 °C

Figure 3 shows the subthreshold emission spectra dynamically changed from electroluminescence to lasing with the increasing current which is measured with the Bruker Vertex 70 FTIR and a nitrogen-cooled HgCdTe detector. The laser spectrum just above threshold indicates that the device operates on the fundamental mode and we can clearly get the stopband of the fundamental mode when the current is 285 mA. From the stopband width Δ*ν* = 3.076 cm^−1^ and the effective index *n*_eff_ = 1/(2νΛ) = 3.153, we calculate a coupling coefficient *κ* = Δ*ν*·*π*·*n*_eff_ = 30.4 cm^−1^, resulting for our HR-coated 2-mm-long cavity in a coupling product *κL* of 12.1, which corresponds well with our device fabrication. The product of *κL* far larger than the previous theoretical investigation *κL* ≈ 1 [20] indicates that an overcoupled scheme is obtained, which is beneficial to secure single mode within the entire current and examined temperature range.

**Fig. 4:**
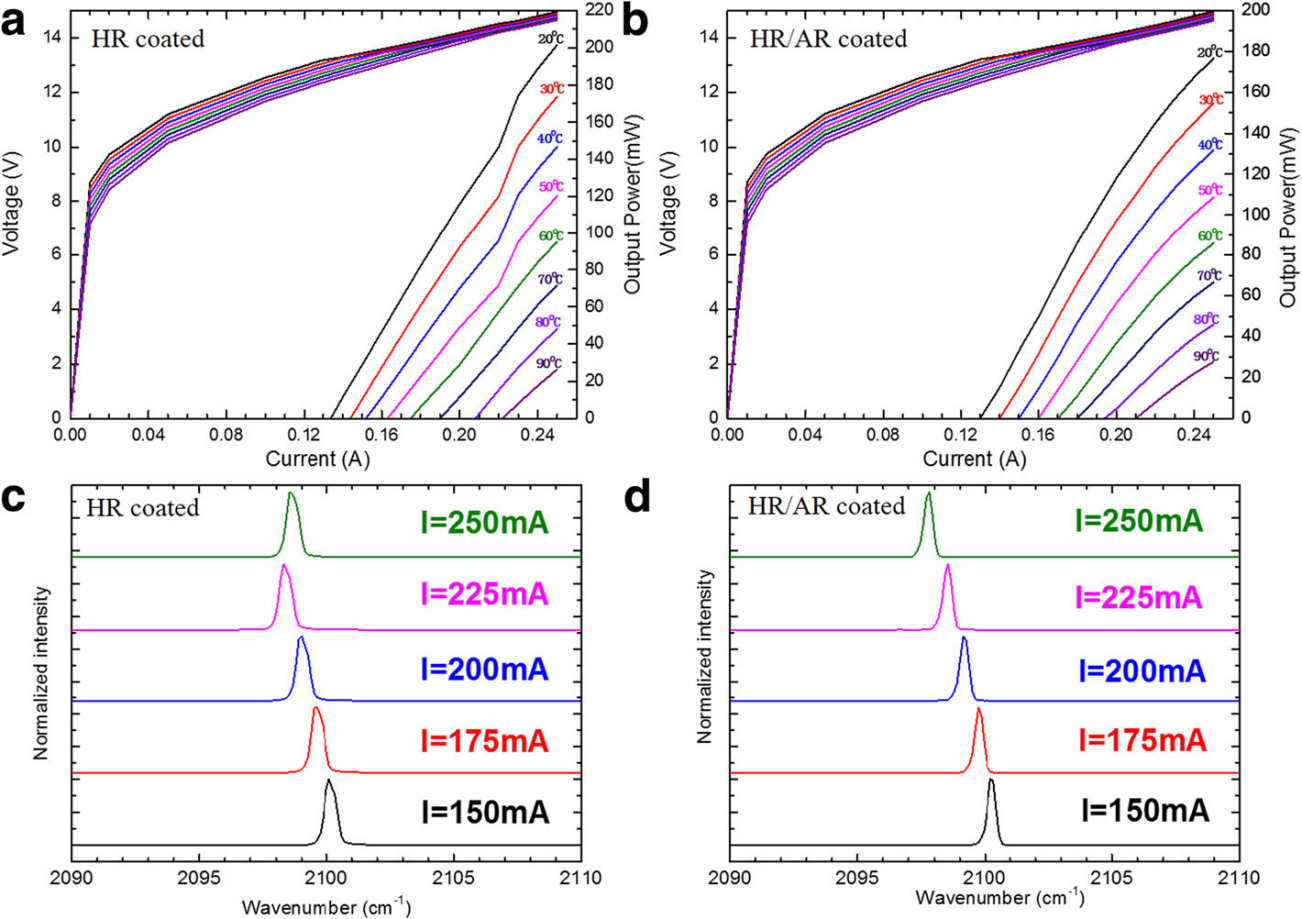
**a**, **b** Output power versus current of the DFB laser operated in CW mode at different heat sink temperatures between 20 and 90 °C along with *V*-*I* curves. **c**, **d** CW lasing spectra at different currents from 150 to 250 mA with a step of 25 mA at 20 °C

Figure 4a shows the typical CW power-current-voltage (*P*-*I*-*V*) curve of the DFB laser at different heat sink temperatures between 20 and 90 °C. The output power reaches 200 mW for the 2-mm-long device with a low threshold current density of 0.65 kA/cm^2^ at 20 °C. The threshold voltage (*V*_th_) of 13.2–14.2 V was measured over the temperature range of 20–90 °C. It is worth noting that mode hop only exists in lower heat sink temperature below 60 °C which can be easily deduced from the *P*-*I* curve. High heat sink temperature would contribute more severe heat accumulation to the laser core so that thermal effect restrained another mode lasing and mode hop would not occur. Figure 4b shows the *P*-*I*-*V* curve of the DFB laser that an antireflection (AR) coating has been deposited on its front facet, and we choose an AR coating reflectivity of 3.4%. Every smooth *P*-*I* curves indicate that there is no mode hop existence all around the temperature we measured. Figure 4c, d shows the lasing spectral at different currents from 150 to 250 mA with a step of 25 mA. It is obvious from Fig. 4d that we achieve a stable single mode around different currents with optimized AR facet coating rather than mode hop in Fig. 4c. The frequency always keeps a linear relation with injection current, and the current tuning coefficient Δ*ν*/Δ*I* = − 0.024 cm^−1^ mA^−1^ proves that AR coating is a simple and efficient method to solve the problem of mode hop in DFB-QCLs.

**Fig. 5:**
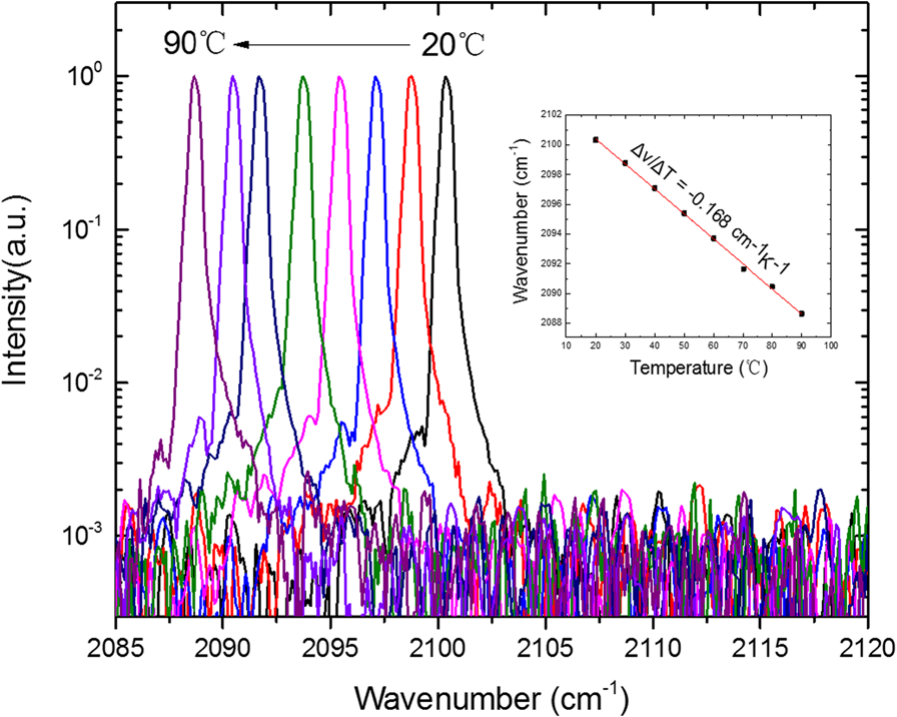
Single-mode emission spectra of the DFB laser at a driving 1.1 threshold current for different heat sink temperatures of 20–90 °C. The insert shows the linearly fit tuning characteristics of the lasing frequency with temperature

Figure 5 shows the emission spectra of the coating DFB laser at different heat sink temperatures from 20 to 90 °C. The measurements were performed using a NICOLET 8700 FTIR spectrometer with 0.25 cm^−1^ resolution in a rapid scan mode. A single longitudinal mode emission is observed among the entire investigated temperature range with a side-mode suppression ratio (SMSR) 26 dB at high temperature of 90 °C. As is shown in the inset of Fig. 5, the peak emission spectrum was observed to shift from 2100.4 cm^−1^ at 20 °C to 2088.6 cm^−1^ at 90 °C, corresponding to a temperature tuning coefficient Δ*ν*/Δ*T* = − 0.168 cm^−1^ K^−1^. The good linear tuning indicated that no mode hopping happened during the change of heat sink temperature. In addition, all mentioned devices display a dominant single lateral far-field under CW operation on the fundamental mode due to the accurate control of ridge width.

**Fig. 6:**
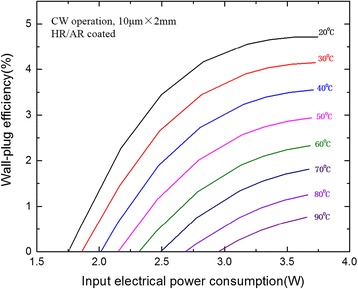
Wall-plug efficiency as a function of the electrical power dissipation for the 2-mm-long HR and AR-coated DFB-QCL

The CW WPE was calculated and plotted as a function of the input electrical power consumption in Fig. 6. At 20 °C, a maximum WPE of 4.7% was obtained around 240 mA with the output power of 170 mW. The maximum WPE were still 2.9 and 0.8% at 50 and 90 °C respectively. To date, these values were still very high for the low-threshold DFB-QCLs due to our high material quality and optimized reflectivity facet coating. It is believed that the WPE can be further improved by the optimized selection of laser cavity lengths considering the coating effect.

## Conclusions

We have demonstrated the room temperature CW operation of single-mode DFB-QCLs at *λ* ~ 4.76 μm. By depositing AR coating consisting of double-layer dielectric Al_2_O_3_ and Ge on front facet, a stable single mode without any mode hop under all currents and temperature conditions has been realized successfully. At 20 °C, CW output power as high as 170 mW has observed with a very low threshold current density of 0.65 kA/cm^2^. Such devices represent an important step towards using stable single-mode operation of DFB-QCLs in mid-infrared spectral range for practical applications.

## Abbreviations

AR: Antireflection
BH: Buried heterostructure
CW: Continuous wave
DFB: Distributed feedback
HR: High reflection
MBE: Molecular beam epitaxy
MOVPE: Metal organic vapor phase epitaxy
PDE: Partial differential equation
PECVD: Plasma-enhanced chemical vapor deposition
*P*-*I*-*V*: Power-current-voltage
QCL: Quantum cascade laser
SMSR: Side-mode suppression ratio
*V*_th_: Threshold voltage
WPE: Wall-plug efficiency
*λ*/4 PS: A quarter-wave phase shift
